# NADPH Oxidase 5 Is Essential for Molting and Oviposition in a Rice Planthopper *Nilaparvata lugens*

**DOI:** 10.3390/insects11090642

**Published:** 2020-09-18

**Authors:** Lu-Yao Peng, Zhen-Wei Dai, Rui-Rui Yang, Zhen Zhu, Wei Wang, Xiang Zhou, Yan-Yuan Bao

**Affiliations:** 1 State Key Laboratory of Rice Biology and Ministry of Agriculture Key Lab of Molecular Biology of Crop Pathogens and Insect Pests, Institute of Insect Sciences, Zhejiang University, Hangzhou 310058, China; luyaopeng@zju.edu.cn (L.-Y.P.); zhenweidai@zju.edu.cn (Z.-W.D.); ruiruiyang@zju.edu.cn (R.-R.Y.); 9961445003@edu.k.u-tokyo.ac.jp (Z.Z.); weiwang2020@zju.edu.cn (W.W.); xiangzhou@zju.edu.cn (X.Z.); 2 Department of Integrated Biosciences, Graduate School of Frontier Sciences, The University of Tokyo, Kashiwa 277-8562, Japan

**Keywords:** *Nilaparvata lugens*, *Nox5*, molting, oviposition, RNAi

## Abstract

**Simple Summary:**

The brown planthopper, *Nilaparvata lugens*, a plant phloem-sucking Hemipteran insect, has become the most destructive pest for rice—the major food source for half of the world’s population. *Nilaparvata lugens* possess robust fecundity, enabling population densities to increase quickly in a favorable environment. *Nilaparvata lugens* has also been used as a model system for ecological studies and for developing effective pest management. To better understand the regulation mechanisms of insect reproduction and to provide insights to improve pest control, we searched *N. lugens* genome and transcriptome databases and identified an *NADPH oxidase 5* gene, which was specifically expressed in ovaries of female adults and freshly laid eggs in rice leaf sheaths. Although homologous *NADPH oxidase 5* genes have been identified in many insect species, their reproductive functions remain unknown. In this work, our findings initially reveal a functional role of the *NADPH oxidase 5* gene in molting and oviposition in *N. lugens*. This novel finding improves our understanding of the reproductive strategies in insects and provides a potential molecular target for effective pest control of the rice planthopper.

**Abstract:**

The brown planthopper *Nilaparvata lugens* is a typical monophagous insect herbivore that feeds exclusively on rice sap. This insect pest causes serious damage to rice crops throughout East Asian countries. Chemical control remains the first choice for managing *N. lugens* populations; however, the use of insecticides has given rise to planthopper resurgence and additional environmental risks. *Nilaparvata lugens* is a model insect of Hemiptera because its whole genome sequence has been elucidated and is susceptible to RNA interference. In this study, our findings revealed that a superoxide-generating gene, *NADPH oxidase 5* (*Nox5*), is essential for molting and oviposition in a Hemipteran insect *Nilaparvata lugens*. Knockdown of *Nox5* transcript levels by RNA interference in 2nd–5th-instar nymphs results in significantly lethal deficits in the molting transitions from nymph–nymph and nymph–adult. *Nox5* knockdown leads to a reduction of hydrogen peroxide in female ovaries and failure of oviposition from the insect ovipositor into the rice leaf sheath. Here, we provide in vivo evidence demonstrating that *Nox5* is a key enzyme for regulating molting and oviposition in this insect species.

## 1. Introduction

The brown planthopper *Nilaparvata lugens* (Hemiptera: Delphacidae), a plant phloem-sucking Hemipteran insect, has emerged as a major pest affecting rice crops in tropical Asia and southern China [1]. *Nilaparvata lugens* possess robust fecundity, enabling population densities to increase quickly in a favorable environment. Once an eruption of this insect pest occurs, little can be done to control the planthoppers [1]. The regulation mechanisms of insect reproduction have attracted great interest for decades. In oviparous insects, fecundity largely depends on oogenesis and oviposition [2]. Oviposition is an important phase for the reproductive success of insects. It has been recognized that some proteins/enzymes play vital roles in insect oviposition through regulating a variety of biological processes. In *Drosophila melanogaster*, matrix metalloproteinase 2 is required for the release of mature oocytes from follicles [3]. Nicotinamide adenine dinucleotide phosphate (NADPH) oxidase plays an essential role in generating superoxide in mature follicles for *Drosophila* oviposition [4]. In *N. lugens*, some progress has been made in understanding its reproductive regulation mechanisms. A mucin-like gene and an eggshell-associated *NlChP38* gene, specifically expressed in the follicular cells, are essential for *N. lugens* oviposition [5,6]. We speculate that there are functional candidate genes contributing to oviposition success, but which remain unexplored. In this study, we searched *N. lugens* genome and developmental stage- and tissue-specific transcriptome databases [7,8,9,10,11] and identified an *NADPH oxidase 5* gene that is specifically expressed in ovaries of female adults and freshly laid eggs in rice leaf sheaths.

NADPH oxidase is an enzyme that primarily catalyzes the production of superoxide from oxygen and NADPH [12]. A family of NADPH oxidases termed the Nox/Duox family has been identified in a wide range of organisms including invertebrates, vertebrates and plants [13]. The mammalian Nox/Duox family contains five Noxes, which have different tissue distribution and biological functions in the generation and regulation of reactive oxygen species (ROS) [14,15,16]. The enzymology and the physiological functions of Noxes have been extensively studied in a variety of mammalian cell types. It is known that NADPH oxidase-produced ROS are involved in immune defense reactions against microbes in mammalian phagocytes [13]. ROS also play an indispensable role in mammalian oviposition [17]. Despite these observations, there is no clear evidence to support an in vivo role of NADPH oxidase-generated ROS in mammalian oviposition. Insects have much fewer *Nox* genes compared with mammals. The genome of *D. melanogaster*, a Dipteran model insect, contains only one *Nox* gene, an ortholog of human *Nox5* [4]. The genome of *Bombyx mori,* a Lepidoptera model organism, encodes two *Nox* genes, which are the orthologs of human *Nox4* and *Nox5*. The *Nox* gene family in insects may have undergone a contraction during the evolutional process. So far, there have been very few in vivo data collected on *Nox* functions in insects. An earlier study proposed the concept that *Drosophila Nox5* regulated oviposition by mediating ovarian smooth muscle contraction [13]. Recent work provided new evidence demonstrating that *Nox5* does not play a major role in ovarian muscle but is essential for female oviposition through generating hydrogen peroxide (H_2_O_2_), which is formed by dismutation of the primary NADPH oxidase product superoxide in *Drosophila* mature follicles [4]. Currently, the genetic evidence supporting *Nox* function in insect oviposition is limited in *D. melanogaster*. Much less is known about *Nox* in other insect species. *Nilaparvata lugens* is a model insect of Hemiptera because its whole genome sequence has been elucidated [10] and it possesses a highly RNAi-sensitive system [18,19]. In this study, by means of a deep search of the *N. lugens* genome and transcriptome databases, we identified two *Nox* sequences, which are the orthologs of human *Nox4* and *Nox5* genes, respectively. Similarly to the human *Nox5* gene with an N-terminal EF-hand calcium-binding domain, *N. lugens Nox5* contains five EF-hand domains at its N-terminus, whereas the *Nox4* genes of human and *N. lugens* lack the EF-hand domains in their sequences. Here, we investigated the in vivo functions of *Nox5* gene by RNA interference in *N. lugens*. Our findings revealed that knockdown of *Nox5* gene expression resulted in development deficits and the failure of oviposition. RNA interference (RNAi) of *Nox5* significantly reduced the production of H_2_O_2_ in the ovaries of *N. lugens* females. This study presents initial in vivo evidence that Hemipteran insects require *Nox5* enzyme for development and egg-laying.

## 2. Materials and Methods

### 2.1. Insects

The *N. lugens* population was originally collected from a rice field in the Huajiachi Campus of Zhejiang University, Hangzhou, China. The insects were reared at 26 °C ± 0.5 °C with 50% ± 5% humidity on rice seedlings (*Oryza sativa* strain Xiushui 134) under a 16:8 h light/dark photoperiod, as previously described [10].

### 2.2. Development-Stage and Tissue Sample Collection

For developmental stage-specific expression analysis of *Nox5* gene, the samples were separately collected from the laid eggs taken from rice leaf sheaths at 24-h intervals, the 1st–4th-instar nymphs at 12-h intervals, the 5th-instar male nymphs and male adults at 24-h intervals, and the 5th-instar female nymphs and female adults at 12-h intervals. For tissue-specific expression analysis, the brain, salivary gland, fat body, gut, ovary, testis, cuticle, wing pad, macropterous wing and brachypterous wing were dissected from the 5th-instar nymphs—male and female adults on ice under a Leica S8AP0 stereomicroscope (Leica Microsystems GmbH, Wetzlar, Germany). To analyze *Nox5* gene expression in each part of the ovaries, the ovipositor, bursa copulatrix, spermatheca and oviduct were individually collected from female adults. The samples were quickly washed in diethylpyrocarbonate-treated NaCl/Pi solution (pH 7.4), as previously described [9].

### 2.3. Developmental Stage- and Tissue-Expression Patterns of Nox5 by Quantitative Real-Time PCR Analysis

Total RNA was extracted from each developmental stage- and tissue-specific sample using RNAiso plus (TaKaRa, Dalian, China). The concentration and quality of the extracted RNA were measured with a NanoDrop 2000/2000 c spectrophotometer (Thermo Fisher Scientific, Bremen, Germany). First-strand cDNA was synthesized using a Hiscript^®^ II QRT SuperMix for qPCR (+gDNA wiper) Kit (Vazyme, Nanjing, China) to remove any contaminating genomic DNA. RNA with no-reverse-transcriptase was used as the no-template control. Quantitative real-time PCR was run on a CFX Connect^TM^ Real-Time System (Bio-Rad, Hercules, CA, USA) using Hieff™ qPCR SYBR Green Master Mix (Yeasen, Shanghai, China) under the following reaction program: an initial denaturation step at 95 °C for 30 s, followed by 35 cycles at 95 °C for 5 s and 55 °C for 30 s. The pair of *Nox5* gene-specific primers (sense 5′-AGCATGCCGTACTGATAGCC-3′ and anti-sense 5′-TGCCTGCTCGATTTCCAGTT-3′) were designed using the Primer Premier 6.0 program based on the *N. lugens* transcriptomic sequences. As the internal controls, expression of the *N. lugens* housekeeping gene for *18S ribosomal RNA*, *RPS11* and *RPS15* (GenBank accession no. JN662398, XP_022188350 and ACN79501) was analyzed. The results were normalized to the expression level of the internal genes. The ∆∆C_t_ method was used to evaluate the quantitative variation in the transcript levels, as described previously [20].

### 2.4. RNA Interference (RNAi)

The double-stranded RNA of *Nox5* was synthesized by in vitro transcription with the PCR-generated DNA template, which was amplified from the *Nox5* sequence using the MEGAscript T7 Transcription Kit (Vazyme, Nanjing, China). A pair of primers containing the T7 promoter were designed as follows: 5′-TAATACGACTCACTATAGGGAGATGCAAGTACCGCTGATGTGT-3′ (sense primer) and 5′-TAATACGACTCACTATAGGGAGACACTCCAATGCCTGTGGCTA-3′ (anti-sense primer). The amplified sequence, around 500 bp long, was cloned into the pMD19-T vector (TaKaRa). *Aequorea victoria* green fluorescent protein (GFP) was used as a negative control. Following transcription, the DNA templates were removed with TURBO DNase (Ambion). The sizes of double-stranded RNA (dsRNA) products were confirmed by electrophoresis on a 1% agarose gel with TAE buffer. The 2nd-, 3rd-, 4th- and 5th-instar nymphs were anesthetized with carbon dioxide for 15–20 s and each instar nymph was microinjected with approximately 250 ng of dsRNA using the FemtoJet Microinjection System (Eppendorf-Netheler-Hinz, Hamburg, Germany). The treated insects were reared on fresh rice seedlings at 26 °C ± 0.5 °C with 50% ± 5% humidity under a 16:8 h light/dark photoperiod. RNAi effects were determined three days after dsRNA-injection, using quantitative real-time PCR. The transcript level changes of the *Nox5* gene were determined by quantitative real-time PCR analysis.

### 2.5. Observation of Oviposition and Egg Hatching

Oviposition and egg hatching experiments were carried out according to Xu et al. [21] and Shen et al. [22]. Briefly, the 5th-instar nymphs were microinjected with ds*Nox5* and ds*GFP*, respectively, and were reared on fresh rice seedlings until emergence. To ensure successful mating, the mating was performed between the newly emerged female and male adults as follows: a single ds*Nox5*-treated female adult was mated with two ds*Nox5*-treated male adults; similarly, a single ds*Nox5*-treated female adult was mated with two ds*GFP*-treated male adults; and a single ds*GFP*-treated female adult was mated with two ds*Nox5*-treated or two ds*GFP*-treated male adults. The mating between female and male adults was performed in a long glass tube containing three-leaf stage fresh rice seedlings (6.5 ± 0.5 cm long) at 26 °C ± 0.5 °C with 50% ± 5% relative humidity under a 16:8 h light/dark photoperiod for 3 days, then the female and male adults were moved. The hatched nymphs in the rice seedlings were observed and counted at 24-h intervals, then were moved out of the rice seedlings 10 days later. Unhatched eggs in rice leaf sheaths were dissected and counted. Biological replicates were carried out for each mating (n = 15 − 20♀×♂). The egg-laying sites in rice leaf sheaths were observed under a microscope and oviposition behavior was recorded by photography.

### 2.6. Measurement of H_2_O_2_ Production

The production of H_2_O_2_ was measured spectrophotometrically with a Varioskan^®^ Flash microplate reader (Thermo Fisher Scientific, Vantaa, Finland) using the Hydrogen Peroxide Assay Kit (Beyotime Biotech, Shanghai, China). In this assay, H_2_O_2_ converts Fe^2+^ to Fe^3+^, which then complexes with xylenol orange dye to yield a purple product having an absorbance maximum at 560 nm, which could be detected by a spectrometer [23]. Ten micrograms of ovaries of female adults were homogenized in 200 µL lysis buffer using a hand-drive tissue homogenizer on ice and centrifuged at 12,000× *g* at 4 °C for 5 min. The protein concentrations of the supernatant were quantified using a Pierce BCA Protein Assay Kit (Thermo Scientific, Rockford, IL, USA). Aliquots of 50 μL of supernatants and 100 μL of test solutions from the Hydrogen Peroxide Assay Kit were added into each well of a 96-well plate and gently mixed to incubate at room temperature for 30 min and measured immediately using a microplate reader at a wavelength of 560 nm (OD_560_). The concentration of H_2_O_2_ was calculated according to the H_2_O_2_ standard curve. Three independent biological replicates were performed.

### 2.7. Analysis of Evolutionary Relationships

The predicted amino acid sequence of *N. lugens* Nox5 was aligned with the orthologs of other insect species from the NCBI database using the CLUSTAL W program. The phylogenic tree was constructed using the full-length amino acid sequences of Nox5 proteins by the Maximum Likelihood program Mega X (http://www.megasoftware.net/). Phylogenetic relationships were determined using bootstrap analysis with 1000 replications.

### 2.8. Statistical Analyses

Data were analyzed by Student’s *t*-test or one-way ANOVA. All statistical analysis and data plots were performed using Prism software. In the figures, statistically significant differences are presented as ** and indicate *p* < 0.01.

### 2.9. Availability of Supporting Data

The *Nox5* sequence was searched against the *N. lugens* genome database deposited in GenBank under accession number AOSB00000000 (BioProject PRJNA177647) and the *N. lugens* transcriptome in the Sequence Read Archive (SRA) database (http://www.ncbi.nlm.nih.gov/sra, SRX023419) using the tBLASTX algorithm with a cut-off E-value of 10^−10^. The conserved domains were determined using the Simple Modular Architecture Research Tool (SMART) (http://smart.embl.de/) and the Conserved Domains database of the National Center for Biotechnology Information (NCBI) website (http://www.ncbi.nlm.nih.gov/Structure/cdd/wrpsb.cgi). The transcript sequence of *N. lugens Nox5* gene has been submitted to NCBI (http://www.ncbi.nlm.nih.gov/). The accession number is MT629918.

## 3. Results

### 3.1. Identification of an NADPH Oxidase 5 Gene in N. lugens

**Figure 1:**
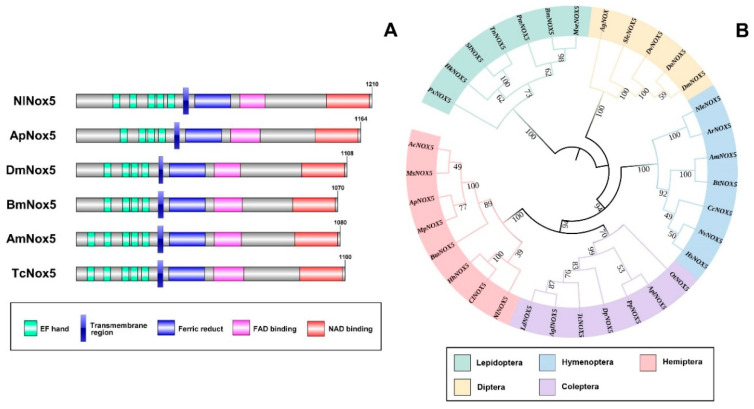
The predicted protein sequence analysis of NADPH oxidase 5 (Nox5) of insects. (**A**) Domain organizations of the deduced Nox5 proteins of several insect species. The conserved domains/motifs were predicted by SMART (http://smart.embl.de/). The green boxes indicate the calcium-binding EF-hand motifs; blue boxes refer to the ferric reductase transmembrane components; pink and red boxes refer to the binding domains for flavine adenine dinucleotide (FAD) and nicotinamide adenine dinucleotide (NAD). Amino acids are numbered on the right of each Nox5 protein. (**B**) Phylogenetic relationship between *Nilaparvata lugens* Nox5 and the orthologs of other insect species. The phylogenetic tree was constructed by Maximum likelihood program Mega X (http://www.megasoftware.net/) with bootstrapping using 1000 replications. The bootstrap values are shown on each internal node. *Ac*, *Aphis craccivora*; *Ms*, *Melanaphis sacchari*; *Ap*, *Acyrthosiphon pisum*; *Mp*, *Myzus persicae*; *Bta*, *Bemisia tabaci*; *Hh*, *Halyomorpha halys*; *Cl*, *Cimex lectularius*; *Nl*, *Nilaparvata lugens*; *Ld*, *Leptinotarsa decemlineata*; *Agl*, *Anoplophora glabripennis*; *Tc*, *Tribolium castaneum*; *Dp*, *Dendroctonus ponderosae*; *Pp*, *Photinus pyralis*; *Apl*, *Agrilus planipennis*; *Ot*, *Onthophagus taurus*; *Hs*, *Harpegnathos saltator*; *Nv*, *Nasonia vitripennis*; *Cc*, *Cephus cinctus*; *Bt*, *Bombus terrestris*; *Am*, *Apis mellifera*; *Ar*, *Athalia rosae*; *Nle*, *Neodiprion lecontei*; *Dm*, *Drosophila melanogaster*; *Do*, *Drosophila obscura*; *Dv*, *Drosophila virilis*; *Sle*, *Scaptodrosophila lebanonensis*; *Ag*, *Anopheles gambiae*; *Mse*, *Manduca sexta*; *Bm*, *Bombyx mori*; *Pm*, *Papilio machaon*; *Tn*, *Trichoplusia ni*; *Sl*, *Spodoptera litura*; *Hk*, *Hyposmocoma kahamanoa*; *Px*, *Plutella xylostella*.

By searching the *N. lugens* genomic and transcriptomic databases, we obtained the complete cDNA sequence of an *NADPH oxidase 5* (*Nox5*). The full-length *Nox5* sequence is 4401 base pair (bp) long and contains an open reading frame of 3633 bp, a 445-bp-long 5′ untranslated region and a 323-bp-long 3′ untranslated region (GenBank accession no. MT629918). The deduced protein consists of 1210 amino acid residues (Figure 1A). Amino acid sequence alignment showed that Nox5 proteins of the different insects contain the characteristic calcium-binding EF-hand motifs at the N-terminus, two transmembrane ferric reductase domains and the binding domains for flavin adenine dinucleotide (FAD) and nicotinamide adenine dinucleotide (NAD) at the C-terminus (Figure 1A). The Nox5 proteins of Hemipteran *N. lugens*, Hymenopteran *Apis mellifera* and Coleopteran *Tribolium castaneum* have five classical EF-hand motifs at their N-terminus, whereas Hemipteran *Acyrthosiphon pisum*, Dipteran *D. melanogaster* and Lepidopteran *B. mori* Nox5 proteins only have four EF-hand motifs (Figure 1A). The phylogenetic analysis revealed that *N. lugens* Nox5 cluster with the orthologs of Hemipteran insect species, which are closely related to the Coleoptera cluster, but are distant from the Hymenopteran, Lepidopteran and Dipteran clusters (Figure 1B).

### 3.2. Temporospatial Expression Patterns of Nox5 Gene in N. lugens

**Figure 2:**
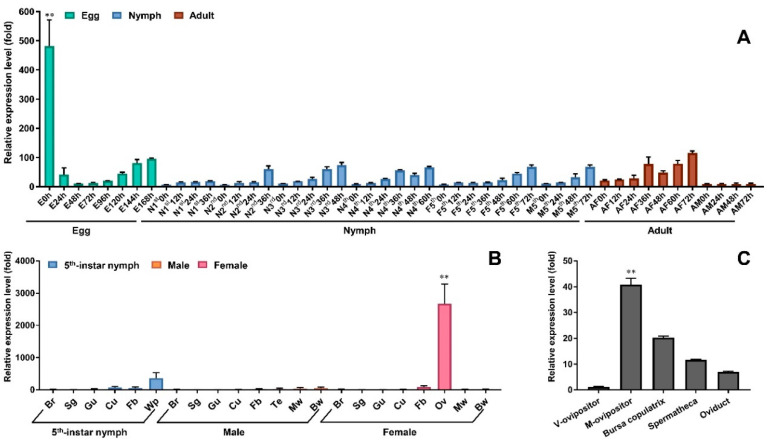
Expression patterns of *Nox5* gene in *N. lugens* by quantitative real-time PCR. (**A**) Developmental expression specificity of *Nox5* gene. Total RNAs were extracted from different developmental stages of *N. lugens* (n = 10–100; namely 100 of the laid eggs at each time point; 20–80 nymphs and 10 adults). E, egg laid in rice leaf sheath; N, nymph; M, male; F, female; A, adult. 1st, 2nd, 3rd, 4th, 5th refer to the 1st-, 2nd-, 3rd-, 4th- and 5th-instar nymphs. (**B**) Tissue expression specificity of *Nox5* gene. Total RNA was individually extracted from the brain, salivary gland, gut, cuticle and fat body of 5th-instar *N. lugens* nymphs, male and female adults (n = 50–100). The wing pad was extracted from 5th-instar nymphs (n = 50); the ovary, testis, macropterous and brachypterous wings (n = 50) were extracted from the female and male adults, respectively. Br, brain; Sg, salivary gland, Gu, gut; Cu, cuticle; Fb, fat body, Wp, wing pad; Te, testis; Ov, ovary, Mw, macropterous wing; Bw, brachypterous wing. (**C**) Ovary-specific expression of *Nox5* gene. Total RNA was extracted from the ovipositor, bursa copulatrix, spermatheca and oviduct of female adults (n = 100). M-ovipositor, the ovipositor of female adults mated with males; V-ovipositor, the ovipositor of virgin females. Quantitative real-time PCR was conducted to analyze the *Nox5* gene expression change. The relative transcript levels of *Nox5* gene in each development stage, each tissue and each part of the ovary were normalized using *N. lugens RPS11*, *RPS15* and *18S rRNA* Ct values as previously described [24]. The reactions were performed with specific primers for amplifying the *Nox5* gene. Three biological replications (mean ± SD) were carried out based on the independent RNA sample and the ΔΔCt method was used to measure the relative transcript levels in each developmental stage and each tissue. The results of triplicate experiments are shown with standard deviations. *p* values are indicated (one-way ANOVA by least significant difference-test). Asterisks (**) indicate significant difference (*p* < 0.01) in the laid eggs at 0 h from other developmental stages; significant difference (*p* < 0.01) in the ovaries of the female adults from other tissues of the 5th instar nymph, male and female adults; significant difference (*p* < 0.01) in M-ovipositor from V-ovipositor, bursa copulatrix, spermatheca and oviduct.

To understand the functional roles of the *Nox5* gene in *N. lugens*, we investigated its expression patterns through developmental stages and tissue distributions using quantitative real-time PCR. *Nox5* transcripts were detected at a notably high level in freshly laid eggs at 0 h in rice leaf sheaths and sharply decreased to a low level in the laid eggs at 24 h, then slowly increased to attach a high level at 168 h, just before egg hatching (Figure 2A). *Nox5* displayed similar expression patterns in the 1st–5th-instar nymphal stages. Its transcripts were detected at very low levels in newly hatched nymphs at 0 h and gradually increased to the peak levels at a timepoint toward the end of each nymphal stage for nymph–nymph and nymph–adult transitions, namely at 36 h for the 1st–2nd-instar nymphs, 48 h for the 3rd-instar nymphs, 60 h for the 4th-instar nymphs and 72 h for the 5th-instar female and male nymphs. In adults, *Nox5* transcripts were at very low levels in males at 0–72 h after emergence, whereas the transcript levels gradually increased from 0 to 72 h in females. The development expression pattern suggested that *Nox5* has important functions in the molting transition between nymph–nymph, nymph–adult and oviposition process.

Tissue-specific analysis displayed that *Nox5* transcripts were at a high level in the wing pad, but at low levels in the brain, salivary gland, gut, fat body and cuticle of the 5th-instar nymphs (Figure 2B). In female adults, the *Nox5* transcripts had a notably high level in the ovary and a low level in the fat body but were almost undetectable in the brain, salivary gland, gut, cuticle, macropterous and brachypterous wings. In male adults, *Nox5* transcripts were almost undetectable or at extremely low levels in all tested tissues (Figure 2B). Tissue-specificity implicated that *Nox5* is of functional importance for female reproduction. A further investigation aiming at the female ovaries showed that *Nox5* had the highest transcript level in the ovipositor of female adults that mated with males. High transcript levels were also detected in the bursa copulatrix followed by the spermatheca and oviduct of the females. Interestingly, a very low transcript level was observed in the ovipositor of virgin females that did not mate with male adults (Figure 2C).

### 3.3. Effect of Nox5 Knockdown on Development of Nymphs

**Figure 3:**
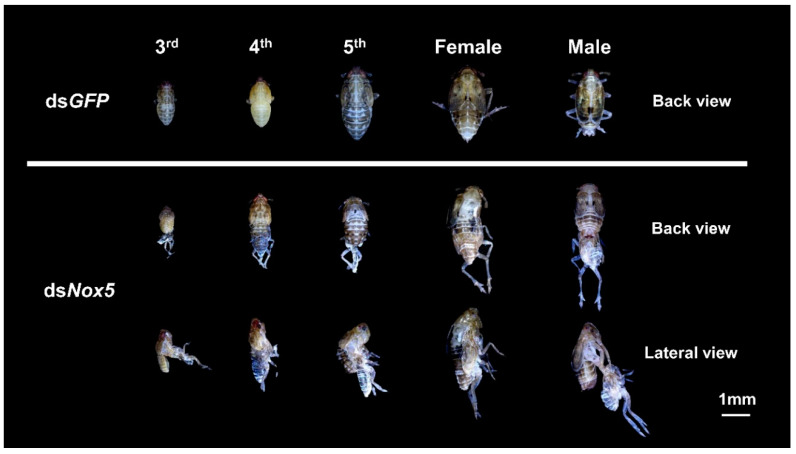
RNA interference (RNAi) analysis of *Nox5* effects on *N. lugens* development. The newly hatched 2nd-, 3rd-, 4th- or 5th-instar nymphs were injected with ds*Nox5*. The 2nd–5th instar nymphs treated with ds*GFP* were used as controls, individually. The lethal phenotypes are shown in ds*Nox5*-injected individuals in the 3rd-, 4th-, 5th-instar nymph and the adult stages.

To understand whether *Nox5* has functional roles during the development process in *N. lugens*, we conducted phenotypic analysis through the RNAi approach at the in vivo level. Knockdown of *Nox5* transcript levels generated lethal defects throughout the nymph and adult stages. The ds*Nox5*-treated nymphs showed a deficiency in the molting 3rd–4th-instar, 4th–5th-instar and 5th-instar–adult transitions (Figure 3). By contrast, ds*GFP*-injected nymphs successfully completed the transitions from nymph–nymph and nymph–adult.

**Figure 4:**
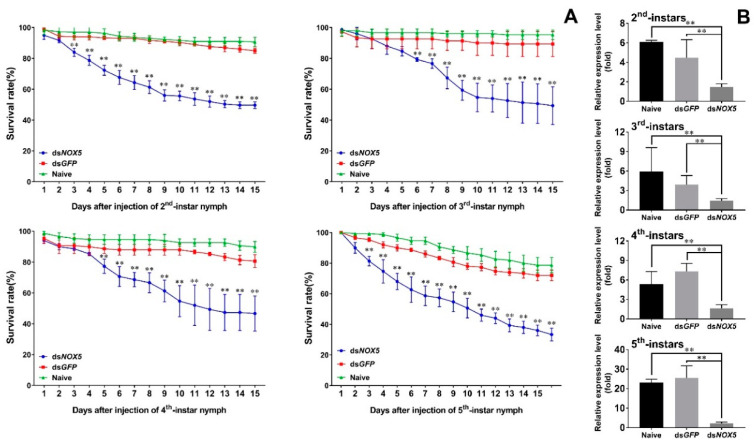
Determination of RNAi effect in *N. lugens* development. (**A**) Dynamic observation of the survival rates in ds*Nox5*-injected *N. lugens* individuals. The newly hatched 2nd-, 3rd-, 4th- or 5th-instar nymphs were individually injected with ds*Nox5*. Non- and ds*GFP*-treated nymphs were used as controls, respectively. The survival rates were calculated from three biological replicates (mean ± SD). For each treatment, n = 100 nymphs. *p* < 0.01 was considered statistically significant (**), different from non- and ds*GFP* treatments. (**B**) The transcript level variations (in fold change) of *Nox5* in 2nd-, 3rd-, 4th- and 5th-instar nymphs were determined by quantitative real-time PCR, as described in Figure 2. Naive and ds*GFP* refer to non- and ds*GFP*-treated controls, respectively. The results of triplicate experiments are shown with standard deviations. *p* values are indicated (Student’s *t*-test). Asterisks (**) indicate significant difference (*p* < 0.01) from controls.

RNAi led to a significant reduction in the survival rates of the insects. The survival rates of ds*Nox5*-treated 2nd-instar nymphs were 84% at 3 d.p.i. (days post injection) and gradually decreased to 49% at 15 d.p.i (Figure 4A). The survival rates of ds*Nox5*-treated 3rd- and 4th-instar nymphs were 88%–92% at 3 d.p.i. and decreased to 46%–49% at 15 d.p.i. The survival rates of ds*Nox5*-treated 5th-instar nymphs were 84% at 3 d.p.i. and quickly declined to 33% at 15 d.p.i. By contrast, the survival rates of non- and ds*GFP*-treated 2nd-, 3rd- or 4th-instar nymphs were more than 85% and the 5th-instar nymphs were more than 75% at 15 d.p.i. Quantitative real-time PCR confirmed that the transcript levels of *Nox5* in each developmental stage (2nd–5th nymphs) were notably reduced in RNAi-treated nymphs compared with non- and ds*GFP*-treated controls (Figure 4B).

### 3.4. Effect of Nox5 Knockdown on Oviposition of Female Adults

**Figure 5:**
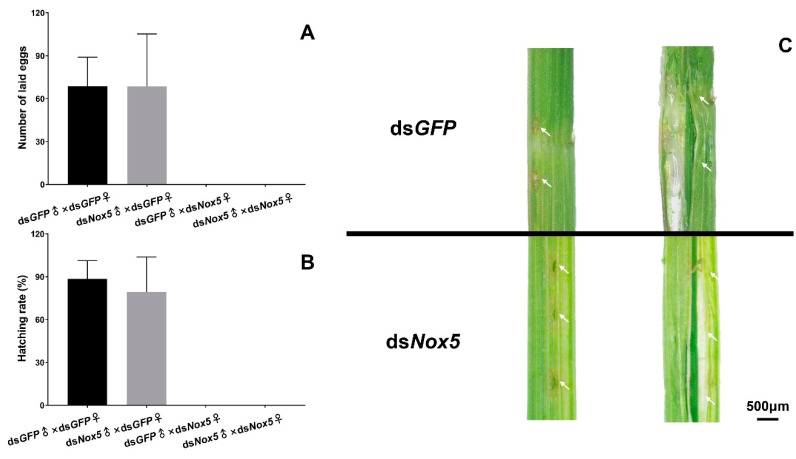
RNAi of *Nox5* effects on egg-laying and hatching. (**A**) Calculation of egg-laying rates. The 5th-instar nymphs were injected with ds*Nox5* and the newly emerged female adults were mated with male adults. The number of eggs laid in rice leaf sheaths from each mating pair was calculated. The *x*-axis indicates each mating pair, namely, ds*GFP*-treated females and males; ds*GFP*-treated females and ds*Nox5*-treated males; ds*Nox5*-treated females and ds*GFP*-treated males; ds*Nox5*-treated females and males. The *y*-axis shows the number of eggs laid from each mating pair. Three biological replicates (mean ± SD) were carried out (n=15–20 ♀×♂). (**B**) Determination of hatching rates. Hatching rates were calculated from the eggs laid in rice leaf sheaths from each mating pair as described above. Three biological replicates (mean ± SD) were carried out (n=15–20 ♀×♂). (**C**) Observation of the eggs laid in the rice leaf sheath. The eggs were dissected from the rice leaf sheath at the 3rd day after oviposition of ds*Nox5*- and ds*GFP*-injected females that mated with male adults. The arrows indicate the egg-laying sites in the rice leaf sheath.

As the *Nox5* gene was highly expressed in the ovaries of female adults and the freshly laid eggs at 0 h in rice leaf sheaths, we investigated the function of *Nox5* in oviposition of female adults using RNAi. No eggs were laid in rice leaf sheaths from ds*Nox5*-injected females that mated with ds*Nox5*-injected males or ds*GFP*-treated male controls (Figure 5A). By contrast, about 60–80 eggs were laid in rice leaf sheaths from each ds*GFP*-treated female that mated with ds*Nox5*-injected males or ds*GFP*-treated male controls and 80%–90% of the laid eggs were successfully hatched into the nymphs (Figure 5B), suggesting that *Nox5* plays a vital role for oviposition of female adults but is not necessary for male reproduction. Subsequently, we investigated the oviposition behavior of female adults. The ds*Nox5*-injected females had oviposition behavior by inserting the ovipositor into rice leaf sheaths and leaving the egg-laying sites but no eggs in rice leaf sheaths (Figure 5C).

**Figure 6:**
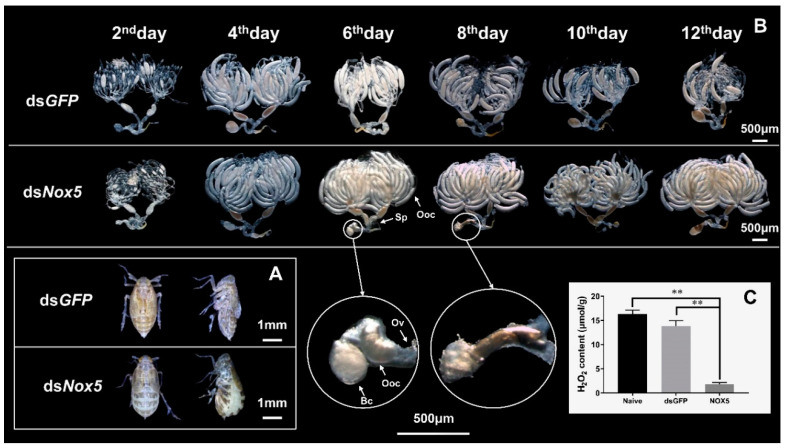
RNAi of *Nox5* effects on oocyte formation in female ovaries. (**A**) Observation of the body change in female adults after RNAi. The 5th-instar nymphs were injected with ds*Nox5* and the female adults that mated with males at the 10th day were used to observe the body morphology. The left and right images show the back and lateral views of ds*GFP*- and ds*Nox5*-injected females, respectively. (**B**) The ovaries were dissected from the ds*Nox5*-treated female adults at the 2nd, 4th, 6th, 8th, 10th and 12th days after mating and were observed under a Leica S8AP0 stereomicroscope. The enlarged circles show the mature oocytes in the lateral oviduct of the female adults. Sp, spermatheca, Ooc, oocyte, Ov, oviduct, Bc, bursa copulatrix. (**C**) The H_2_O_2_ content in the ovaries of ds*Nox5*-treated female adults at the 8th day after mating was determined by means of a Hydrogen Peroxide Assay Kit (Beyotime Biotech, Shanghai, China). Naive and ds*GFP*-treated insects were used as controls.

Furthermore, we observed that ds*Nox5*-treated female adults displayed an obviously swollen abdomen and the stretched intersegmental membranes in the tergum at the 12th day after mating with males, whereas the female controls completed oviposition and showed flat abdomens at the 12th day after mating with males (Figure 6A). We dissected the ovaries of ds*Nox5*-injected female adults that mated with ds*GFP*-treated males to calculate the number of mature oocytes and to observe the morphological changes of oocytes. Reduction in the number and malformation of oocytes was not observed in the ovaries of ds*Nox5*-injected females from the 4th to the 12th days after mating with males. Generally, *N. lugens* female oviposition begins at the 3rd–4th days after mating and egg-laying is completed around the 8th–12th days, at which time a few mature oocytes remain in the ovaries (Figure 6B). However, in ds*Nox5*-injected females, the ovaries were full of banana-shaped mature oocytes at the 4th–12th days after mating. Interestingly, we found a mature oocyte located at the lateral oviduct in a ds*Nox5*-treated female individual, but this oocyte could not be laid in the rice leaf sheaths (Figure 6B). Video indicated that the ds*Nox5*-treated females tried their best to lay eggs from the ovipositor onto rice leaf sheaths but failed, while ds*GFP*-treated females successfully laid eggs on the rice leaf sheaths (Supplementary Video 1). To understand whether ds*Nox5* treatment influences ROS production, we measured H_2_O_2_ content in the ovaries of females at the 8th day after mating. *Nox5* knockdown led to a significant reduction of H_2_O_2_ content by 87% and 89% in the ovaries of ds*Nox5*-injected females compared with those of ds*GFP*-treated and non-treated female controls, respectively (Figure 6C), suggesting that *Nox5* is important for H_2_O_2_ production.

## 4. Discussion

In this study, we investigated the in vivo function of the *NADPH oxidase* gene, *Nox5*, in a Hemipteran model insect, *N. lugens*. The deduced amino acid sequence of the *Nox5* gene contains the characteristic domains, including calcium-binding EF-hand motifs, transmembrane components and FAD- and NAD-binding domains, which are highly conserved in the orthologs of many insect species and mammals, implying its primary functions in the production of superoxide and the regulation of intracellular calcium flux. Here, our findings revealed that *Nox5* played essential roles in the development and oviposition of *N. lugens*. Knockdown of *Nox5* transcript levels in each nymphal stage led to significantly lethal deficits. The nymphs treated with ds*Nox5* hardly completed the molting transition of 3rd–4th-instar, 4th–5th-instar and 5th-instar–adult, suggesting that *Nox5* has important functions in molting and development mechanisms in this insect species.

Tissue specificity indicated that the *Nox5* gene had the highest transcript level in the ovaries among all tested tissues of the 5th-instar nymphs and adults. A further investigation on the ovaries revealed that the *Nox5* gene had a much higher transcript level in the ovipositor than in other parts, including the bursa copulatrix, spermatheca and oviduct, in the mated females. Moreover, the *Nox5* gene showed a notably higher transcript level in the ovipositor of the mated females than the virgin females without mating, implying the functional importance of the *Nox5* gene for the oviposition of the fertilizable oocytes in ovipositor. Knockdown of *Nox5* by RNAi did not reduce the number of mature oocytes in female ovarioles, nor did it cause morphologic changes in mature oocytes, implicating that this enzyme may not be required for oocyte formation and maturation in the ovaries. In *N. lugens*, the female adults generally lay the mature oocytes at the 3rd–4th days after mating, and a few mature oocytes remain in the ovaries around the 8th–12th days after mating with male adults. However, ds*Nox5*-treated females retained many mature oocytes in the ovaries at the 12th day after mating with either ds*Nox5*-injected or ds*GFP*-treated male adults. Furthermore, we observed that the female adults showed abnormally expanded abdomens with accumulated oocytes in the ovaries. These females displayed apparent oviposition behavior by inserting the ovipositor into rice leaf sheaths. We found that knockdown of *Nox5* did not affect the release of the mature oocytes from ovarioles to the lateral oviduct, but inhibited the laying of the oocytes from the ovipositor into rice leaf sheaths, which led to the failure of oviposition. By contrast, ds*Nox5*-treated males that mated with ds*GFP*-treated females successfully laid the eggs in rice leaf sheaths, and around 80% of the laid eggs hatched into nymphs. These observations indicated that *Nox5* plays a determinant role in female oviposition but is not essential for male reproduction.

The primary function of NADPH oxidase is to produce ROS by transferring an electron from NADPH to oxygen. ROS are a collection of highly reactive molecules containing molecular oxygen (O_2_), superoxide anion (O_2_^•−^), hydrogen peroxide (H_2_O_2_) and hydroxyl radicals (^•^OH) [25]. In insects, ROS are involved in immune defense reactions against a broad range of pathogens. Nevertheless, little is known about the role of ROS in insect oviposition. For oviparous insects, the fertilizable oocytes are released from the ovipositor into the external environment. During this process, ROS production may be necessary for successful oviposition and survival of the newly laid eggs. In the present study, to understand the reasons for oviposition failure, we investigated ROS generation in female ovaries by RNAi. Because of the diversity of ROS and their intrinsic instability, we mainly measured H_2_O_2_ levels in ovaries. Knockdown of *Nox5* significantly reduced H_2_O_2_ content in female ovaries, suggesting that *Nox5* is involved in the production of H_2_O_2_. The *Nox5* gene had a notably high transcript level in the laid eggs at a particular time point (0 h) and rapidly decreased levels at 24 h after egg-laying in rice leaf sheaths, implicating a possibility that *Nox5*-generated ROS are required for oviposition of mature oocytes from the ovipositor to rice tissue and for the survival of freshly laid eggs to overcome new environmental challenges, i.e., defenses from plant hosts and pathogenic infection. Ritsick et al. reported that *Drosophila Nox* modulates intracellular calcium flux via H_2_O_2_ [13]. As *N. lugens* Nox5 contains five calcium-binding EF-hand motifs, we hypothesize another possibility—that *Nox5* regulates calcium flux in the ovaries of female adults. The detailed regulation mechanisms of the *Nox5* gene will need to be further elucidated. The knowledge of in vivo function of the *Nox* gene in molting and metamorphosis mechanisms is very limited in insects. In this study, the observation of the lethal phenotypes in ds*Nox5*-treated nymphs, combined with the expression patterns of *Nox5* in nymphs with the lowest levels at 0 h and the highest levels at the end of each instar, imply that *Nox*-generated ROS are essential for molting transition and the survival of newly hatched nymphs or emerged adults when encountering the external environment. Further studies investigating the actions of *Nox*-associated ROS and calcium flux will be required to understand development regulation mechanisms in insects.

## 5. Conclusions

In conclusion, our study revealed that *Nox5* plays an important role in the molting transition from nymph–nymph and nymph–adult in *N. lugens*. *Nox5* is a key enzyme in the oviposition process of the mature oocytes from the *N. lugens* ovipositor to rice leaf sheaths. Our findings provide valuable information for a better understanding of the biological roles of *Nox*-generated ROS in development and oviposition mechanisms in insect species. This work will be useful for producing a potential target gene for the management of rice pests in the future.

## Supplementary Materials

The following are available online at https://www.mdpi.com/2075-4450/11/9/642/s1, Video S1: Observation of oviposition behavior of *N. lugens* female adults. The insertion of ovipositor into rice leaf sheaths was seen in ds*GFP*- and ds*Nox5*-treated females, respectively.

## References

[1] Bao Y.Y., Zhang C.X. (2019). Recent advances in molecular biology research of a rice pest, the brown planthopper. J. Integr. Agric..

[2] Minkenberg O., Tatar M., Rosenheim J.A. (1992). Egg load as a major source of variability in insect foraging and oviposition behavior. Oikos.

[3] Deady L.D., Shen W., Mosure S.A., Spradling A.C., Sun J. (2015). Matrix metalloproteinase 2 is required for ovulation and corpus luteum formation in *Drosophila*. PLoS Genet..

[4] Li W., Young J.F., Sun J. (2018). NADPH oxidase-generated reactive oxygen species in mature follicles are essential for *Drosophila* ovulation. Proc. Natl. Acad. Sci. USA.

[5] Lou Y.H., Shen Y., Li D.T., Huang H.J., Lu J.B., Zhang C.X. (2019). A mucin-like protein is essential for oviposition in *Nilaparvata lugens*. Front. Physiol..

[6] Lou Y.H., Lu J.B., Li D.T., Ye Y.X., Luo X.M., Zhang C.X. (2019). Amelogenin domain-containing NlChP38 is necessary for normal ovulation in the brown planthopper. Insect Mol. Biol..

[7] Bao Y.Y., Wang Y., Wu W.J., Zhao D., Xue J., Zhang B.Q., Shen Z.C., Zhang C.X. (2012). De novo intestine-specific transcriptome of the brown planthopper *Nilaparvata lugens* revealed potential functions in digestion, detoxification and immune response. Genomics.

[8] Bao Y.Y., Qu L.Y., Zhao D., Chen L.B., Jin H.Y., Xu L.M., Cheng J.A., Zhang C.X. (2013). The genome- and transcriptome-wide analysis of innate immunity in the brown planthopper, *Nilaparvata lugens*. BMC Genom..

[9] Bao Y.Y., Qin X., Yu B., Chen L.B., Wang Z.C., Zhang C.X. (2014). Genomic insights into the serine protease gene family and expression profile analysis in the planthopper, *Nilaparvata lugens*. BMC Genom..

[10] Xue J., Zhou X., Zhang C.X., Yu L.L., Fan H.W., Wang Z., Xu H.J., Xi Y., Zhu Z.R., Zhou W.W. (2014). Genomes of the rice pest brown planthopper and its endosymbionts reveal complex complementary contributions for host adaptation. Genome Biol..

[11] Xue J., Bao Y.Y., Li B.l., Cheng Y.B., Peng Z.Y., Liu H., Xu H.J., Zhu Z.R., Lou Y.G., Cheng J.A. (2010). Transcriptome analysis of the brown planthopper *Nilaparvata lugens*. PLoS ONE.

[12] Babior B.M. (2004). NADPH oxidase. Curr. Opin. Immunol..

[13] Ritsick D.R., Edens W.A., Finnerty V., Lambeth J.D. (2007). Nox regulation of smooth muscle contraction. Free Radic. Biol. Med..

[14] Bedard K., Krause K.H. (2007). The NOX family of ROS-generating NADPH oxidases: Physiology and pathophysiology. Physiol. Rev..

[15] Brown D.I., Griendling K.K. (2009). Nox proteins in signal transduction. Free Radic. Biol. Med..

[16] Lambeth J.D. (2004). Nox enzymes and the biology of reactive oxygen. Nat. Rev. Immunol..

[17] Shkolnik K., Tadmor A., Ben-Dor S., Nevo N., Galiani D., Dekel N. (2011). Reactive oxygen species are indispensable in ovulation. Proc. Natl. Acad. Sci. USA.

[18] Xu H.J., Chen T., Ma X.F., Xue J., Pan P.L., Zhang X.C., Cheng J.A., Zhang C.X. (2013). Genome-wide screening for components of small interfering RNA (siRNA) and micro-RNA (miRNA) pathways in the brown planthopper, *Nilaparvata lugens* (Hemiptera: Delphacidae). Insect Mol. Biol..

[19] Zhou X., Ye Y.Z., Ogihara M.H., Takeshima M., Fujinaga D., Liu C.W., Zhu Z., Kataoka H., Bao Y.Y. (2020). Functional analysis of ecdysteroid biosynthetic enzymes of the rice planthopper, *Nilaparvata lugens*. Insect Biochem. Mol. Biol..

[20] Huang H.J., Liu C.W., Huang X.H., Zhou X., Zhuo J.C., Zhang C.X., Bao Y.Y. (2016). Screening and functional analyses of *Nilaparvata lugens* salivary proteome. J. Proteome Res..

[21] Xu L., Huang H.J., Zhou X., Liu C.W., Bao Y.Y. (2017). Pancreatic lipase-related protein 2 is essential for egg hatching in the brown planthopper, *Nilaparvata lugens*. Insect Mol. Biol..

[22] Shen Y., Chen Y.Z., Lou W.H., Zhang C.X. (2019). Vitellogenin and vitellogenin-like genes in the brown planthopper. Front. Physiol..

[23] Wei G., Lai Y.L., Wang G.D., Chen H., Li F., Wang S.B. (2017). Insect pathogenic fungus interacts with the gut microbiota to accelerate mosquito mortality. Proc. Natl. Acad. Sci. USA.

[24] Zhou X., Peng L.Y., Wang Z.C., Wang W., Zhu Z., Huang X.H., Chen L.B., Song Q.S., Bao Y.Y. (2019). Identification of novel antimicrobial peptides from rice planthopper, *Nilaparvata lugens*. Insect Biochem. Mol. Biol..

[25] Lam P.L., Wong M.M., Hung L.K., Yung L.H., Tang J.C.O., Lam K.H., Chung P.Y., Wong W.Y., Ho Y.W., Wong R.S.M. (2020). Miconazole and terbinafine induced reactive oxygen species accumulation and topical toxicity in human keratinocytes. Drug Chem. Toxicol..

